# The RNA uridyltransferase Zcchc6 is expressed in macrophages and impacts innate immune responses

**DOI:** 10.1371/journal.pone.0179797

**Published:** 2017-06-30

**Authors:** Elyse Kozlowski, Gregory A. Wasserman, Marcos Morgan, Dónal O’Carroll, Nora-Guadalupe P. Ramirez, Suryaram Gummuluru, Jasmine Y. Rah, Adam C. Gower, Michael Ieong, Lee J. Quinton, Joseph P. Mizgerd, Matthew R. Jones

**Affiliations:** 1 Pulmonary Center, Boston University School of Medicine, Boston, Massachusetts, United States of America; 2 Department of Medicine, Boston University School of Medicine, Boston, Massachusetts, United States of America; 3 Department of Microbiology, Boston University School of Medicine, Boston, Massachusetts, United States of America; 4 European Molecular Biology Laboratory (EMBL), Mouse Biology Unit, Monterotondo, Italy; 5 MRC Centre for Regenerative Medicine, Institute for Stem Cell Research, School of Biological Sciences, University of Edinburgh, Edinburgh, United Kingdom; 6 Clinical and Translational Science Institute, Boston University School of Medicine, Boston, Massachusetts, United States of America; 7 Department of Pathology and Laboratory Medicine, Boston University School of Medicine, Boston, Massachusetts, United States of America; 8 Department of Biochemistry, Boston University School of Medicine, Boston, Massachusetts, United States of America; Louisiana State University, UNITED STATES

## Abstract

Alveolar macrophages orchestrate pulmonary innate immunity and are essential for early immune surveillance and clearance of microorganisms in the airways. Inflammatory signaling must be sufficiently robust to promote host defense but limited enough to prevent excessive tissue injury. Macrophages in the lungs utilize multiple transcriptional and post-transcriptional mechanisms of inflammatory gene expression to delicately balance the elaboration of immune mediators. RNA terminal uridyltransferases (TUTs), including the closely homologous family members Zcchc6 (TUT7) and Zcchc11 (TUT4), have been implicated in the post-transcriptional regulation of inflammation from studies conducted *in vitro*. *In vivo*, we observed that Zcchc6 is expressed in mouse and human primary macrophages. Zcchc6-deficient mice are viable and born in Mendelian ratios and do not exhibit an observable spontaneous phenotype under basal conditions. Following an intratracheal challenge with *S*. *pneumoniae*, Zcchc6 deficiency led to a modest but significant increase in the expression of select cytokines including IL-6, CXCL1, and CXCL5. These findings were recapitulated *in vitro* whereby Zcchc6-deficient macrophages exhibited similar increases in cytokine expression due to bacterial stimulation. Although loss of Zcchc6 also led to increased neutrophil emigration to the airways during pneumonia, these responses were not sufficient to impact host defense against infection.

## Introduction

Innate immunity is essential for host protection against pathogens. As an integral member of pulmonary innate defense, the alveolar macrophage is a first-responder cell type critical to initiating host immune responses during infection. Resident alveolar macrophages are long-lived and function to clear inhaled and cellular debris, and trigger innate defenses upon detection of microbial products [1–3]. As one of the first cells encountering inhaled particles and pathogens [4], macrophages can initiate and maintain a delicately balanced series of immune responses robust enough to prevent infection but without injurious inflammation. Transcriptional and post-transcriptional regulation of cytokines and other inflammatory mediators is an important means through which immune responses are coordinated to be effective yet appropriate [5–9].

As integral contributors to post-transcriptional mechanisms of RNA regulation [2, 10–17], RNA terminal uridyltransferases (TUTs) play widespread roles established from *in vitro* models, but their integrated functions in mammalian biology and homeostasis remain largely speculative. We previously reported a role for the RNA terminal uridyltransferase (TUT) enzyme Zcchc11 (TUT4) in the post-transcriptional regulation of the inflammatory cytokine IL-6 [18]. This enzyme contributes to the expression of IL-6 and growth factors by hepatocytes in young and rapidly growing mice [19], but otherwise the functional significance of Zcchc11 appears to be minimal. Zcchc11 has been reported to function in stem cell biology and is essential to the *in vitro* maintenance of cellular pluripotency [14, 20], but such roles have yet to be observed *in vivo*. The closest mammalian TUT homolog to Zcchc11 is Zcchc6 (TUT7). Zcchc6 uridyltransferase can uridylate the same miRNAs as Zcchc11 and shows overlapping and somewhat redundant roles in the *in vitro* maintenance of pluripotent stem cells [21] [22]. However, Zcchc6 is capable of uridylating a wide variety of miRNAs [22–24] and even mRNAs [25], suggesting additional biological functions downstream of this enzyme. To our knowledge, the present studies, focused on the influence of Zcchc6 in mice with and without pneumonia, constitute the first to report consequences of a Zcchc6 deficiency *in vivo*.

## Experimental procedures

### Ethics statement

All animal studies were performed in accordance with U.S. Federal Law, and approved by the Boston University School of Medicine Institutional Animal Care and Use Committee (IACUC) (Permit #14859). Animals were anesthetized and euthanized using an approved protocol and all efforts were made to minimize suffering.

### Mice

All murine studies were performed under approval of the Boston University School of Medicine Institutional Animal Care and Use Committee (IACUC). Mice were maintained under pathogen free conditions with access to food and water *ad libitum*. All experiments were performed using both male and female mice at 8–16 weeks of age. Experiments using non-genetically modified animals were conducted with C57BL/6 mice purchased from Jackson Laboratories (Bar Harbor, ME). The Zcchc6-floxed model was generated by flanking the critical exons 16 and 17 of the *Zcchc6* gene with *loxP* sites using homologous recombination (Dr. O’Carroll, manuscript in preparation) so that Cre recombinase-mediated excision leads to a frame-shift mutation to elicit nonsense-mediated decay of the mutant transcript. To create Zcchc6-deficient mice, these mice were bred with the B6.FVB-Tg(EIIa-cre)C5379Lmgd/J mouse (Jackson Laboratories # 003724), which harbors a *cre* transgene under control of the ubiquitous EIIa promoter and leads to mutation of floxed alleles in the germline. Selective breeding established *Zcchc6*^***+/-***^ heterozygotes which lacked the Cre transgene, thereafter bred together to derive Zcchc6-deficient *Zcchc6*^**-/-**^ mice.

### Primary human cell isolation and culture

For primary human macrophages, bronchoalveolar lavage (BAL) was performed on healthy, non-smoking volunteers in accordance with an informed consent protocol approved by Institutional Review Board of Boston University Medical Center as previously conducted [26]. Alveolar macrophages were isolated by adherence to plastic. Non-adherent cells were removed by washing and the remaining adherent cells averaged >98% viability as verified by trypan blue exclusion. Primary human monocytes were isolated as previously described [27]. Peripheral blood mononuclear cells (PBMCs) were isolated from peripheral blood of healthy donors. CD14^+^ monocytes were isolated from PBMCs using CD14-coated magnetic beads (Miltenyi Biotech), and cell purity was assessed to be >95% by FACS. The human monocyte cell lines U937 and THP-1 were obtained from the American Type Culture Collection (ATCC). For the differentiation experiment, cells were seeded in 6-well tissue culture dishes, stimulated with 20 ng/mL (U937) or 5 ng/mL (THP-1) Phorbol 12-myristate 13-acetate (PMA; Sigma Aldrich P8139) overnight, and collected the next day in protein lysis buffer.

### Murine bronchoalveolar and pleural lavage

Lungs were lavaged with ice-cold PBS 10 times in 1 mL increments as previously described [26, 28]. Isolated cells were centrifuged at 300 x *g* for 5 minutes at 4°C, then cytocentrifuged and stained with Diff-Quick (Dade-Behring) to perform cell differential analysis. Total RNA was prepared using Qiazol, and purified using an RNAeasy column (Qiagen). To isolate macrophages from the pleural cavities, mice were sacrificed and the pleural space was lavaged as previously described [29]. In short, after euthanasia, the thoracic cavity is exposed and a small incision is inserted into the dorsal tip of the diaphragm. Using a 1 mL sterile Pasteur pipet, the pleural cavity is lavaged 8 times with 1 mL of ice-cold RPMI 1640 medium supplemented with 1X penicillin/streptomycin and 2 mM L-glutamine. Isolated cells were centrifuged at 300 x *g* for 5 minutes at 4°C, then cytocentrifuged and stained with Diff-Quick (Dade-Behring) to perform cell differential analysis.

### Murine bone marrow macrophage isolation, culture and stimulation

Murine bone marrow macrophages were isolated as previously described [30]. For the differentiation time course, macrophages were collected from C57BL/6 mice and seeded onto 10 cm^2^ petri dishes. Cells were collected in Cell Stripper solution (Corning, 25-056-CI) at specified times of adherence under cell culture conditions and lysed using protein lysis buffer. For the stimulation experiments, cells were seeded onto petri dishes with RPMI, 10% fetal bovine serum, and 20% L929 supernatant and allowed to differentiate into macrophages over 7 days as previously described [30, 31]. For stimulations, adherent macrophages were removed with Cell Stripper solution and subsequently plated on non-adherent 6-well plates at a density of 1x10^6^ cells/well. The media was replaced with RPMI containing 10% FBS and supplemented with vehicle or *S*. *pneumoniae*. Bacteria were removed after 2 hours by washing cells with antibiotic containing complete media. Supernatants were collected 4 hours after washing and CXCL1 concentrations were quantified in cellular supernatants by DuoSet ELISA (R&D Systems).

### Protein Isolation and immunoblotting

For protein measurements, cells and tissues were snap frozen in liquid nitrogen for cryostorage at -80°C. Cellular lysis and protein extraction was performed using lysis buffer containing 20mM Tris-HCl pH 7.4, 150mM NaCl, 1mM MgCl_2_, 0.5% NP-40. Total protein content was quantified using the bicinchoninic acid (BCA) assay (Sigma). For all Western blots, total protein was resolved through 3–8% Tris-Acetate polyacrylamide gels (Life Technologies) and transferred to a PVDF membrane (Immobilon) using the NuPAGE blotting system (Invitrogen). The mouse reactive Zcchc6 antibody was obtained from Proteintech (25196-1-AP). The human reactive Zcchc6 antibody was obtained from Sigma Aldrich (HPA020615). An anti-rabbit, HRP-conjugated secondary antibody (Cell Signaling Technology #7074S) followed by ECL chemiluminescence (GE Healthcare, RPN2232) was used for protein detection.

### Quantitative RT-PCR analysis

Total RNA was isolated following the RNAeasy kit protocol (Qiagen). mRNA expression was measured using the TaqMan RNA-to-Ct 1-Step kit (Life Technologies). Primers specific for CXCL1 CXCL2, CXCL5, IL-6, TNF and 18s ribosomal RNA were synthesized by Integrated DNA Technologies and have been described previously [28]. The murine Zcchc6 primer/probe set was obtained from Applied Biosystems (#1189979). All qRT-PCR assays were performed on a real-time PCR machine (Applied Biosystems) using 10 ng of total RNA. For each mRNA, fold induction was normalized to the content of 18S rRNA and expressed as fold-induction relative to a control group.

### Microarray analysis

Total RNA was isolated from alveolar macrophages as described above and quality assessed by using an Agilent Bioanalyzer. Affymetrix GeneChip Mouse Gene ST 2.0 arrays were used to determine transcriptomic profiles. Microarray analysis and quantitative assessment were performed by the Boston University Medical Campus Microarray and Sequencing Resource. CEL files were normalized to produce gene-level expression values using the implementation of the Robust Multiarray Average (RMA) [32] in the *affy* package (version 1.36.1)[33] included within in the Bioconductor software suite (version 2.11) and an Entrez Gene-specific probeset mapping (version 17.0.0) from the Molecular and Behavioral Neuroscience Institute (Brainarray) at the University of Michigan. Array quality was assessed by computing Relative Log Expression (RLE) and Normalized Unscaled Standard Error (NUSE) using the *affyPLM* Bioconductor package (version 1.34.0) [34]. Moderated t tests were performed using the *limma* package (version 3.14.4). Correction for multiple hypothesis testing was accomplished using the Benjamini-Hochberg false discovery rate (FDR) [35]. Analyses were performed using the R environment for statistical computing (version 2.15.1). Raw data are available at the NCBI Gene Expression Omnibus archive with the accession GSE98222.

### Experimental pneumonia

For lung infections, mice were anesthetized by intraperitoneal injection with a 50 mg/kg ketamine and 5 mg/kg xylazine solution. Tracheas were surgically exposed and cannulated using an angiocatheter. *Streptococcus pneumoniae* serotype 19F (Sp19, strain EF3030, provided by Dr. M. Lipsitch, Harvard School of Public Health) or *Escherichia coli* were intratracheally instilled into the left lung lobe. Target instillations of 5 x 10^6^ CFU/mL (Sp19) or 2 x 10^6^ CFU/mL (*E*.*coli*) or were subsequently verified by serial dilutions of the input on 5% sheep blood agar plates.

### Statistical analysis

Statistical analyses were determined using GraphPad Prism (GraphPad Software). The statistical test used is denoted in the figure legend. A *P*-value of less than 0.05 was considered significant. For microarray studies, false discovery rate was used to correct for multiple comparisons.

## Results

### Zcchc6 is expressed in adult lungs and alveolar macrophages

**Fig 1:**
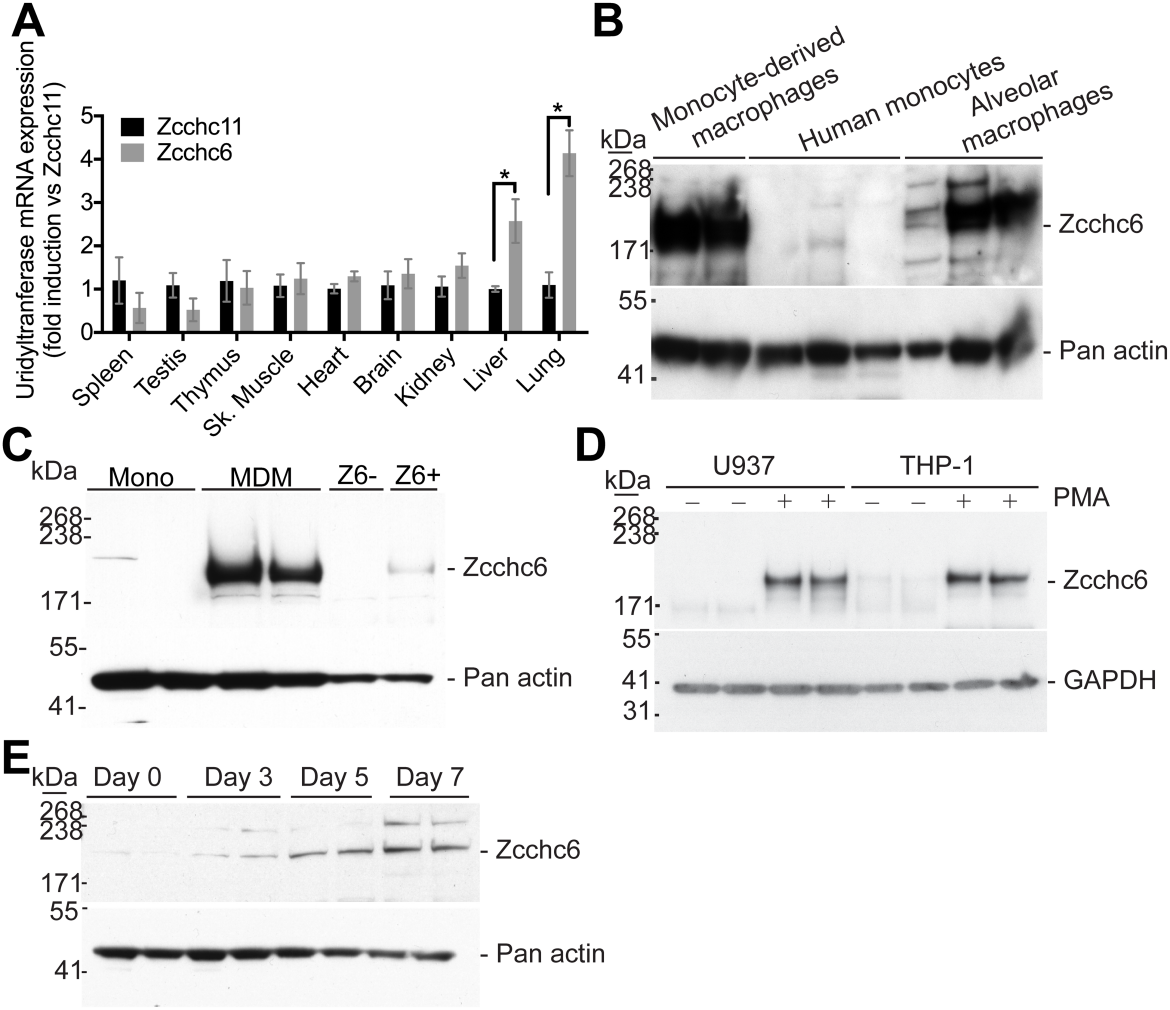
Zcchc6 and Zcchc11 expression in adult mouse tissues. (A) Total RNA was prepared from C57BL/6 mouse tissues and was assayed for Zcchc6 and Zcchc11 mRNA expression by qRT-PCR. Data are normalized to 18S rRNA and displayed as fold induction over Zcchc11 expression (n = 3,3). *p<0.05 ***p<0.001 by two-way ANOVA with Bonferroni post-test. (B)(C) Zcchc6 protein expression was measured by immunoblot in primary human monocyte derived macrophages (4 donors), human monocytes (5 donors), and human alveolar macrophages (3 donors). Lung lysates generated from WT or Z6 deficient mouse lungs were used as a control in (C). Zcchc6 protein expression was assessed in (D) PMA or vehicle treated U937 or THP-1 human monocytic cell lines and in (E) mouse bone marrow-derived macrophages during a 7-day time-course of differentiation.

Previous reports on Zcchc6 and Zcchc11 expression have suggested that both proteins are developmentally regulated and undetectable in adult tissues and organs [24]. At first thought, these findings are clearly anticipated given that abundant levels of mature let-7 microRNA family members are positively associated with differentiated tissue [36] and that Zcchc6 and Zcchc11 are negative regulators of let-7 [21]. However, both enzymes have been detected in at least some cells and tissues of adult animals [18, 19, 24]. The degree to which each TUT is differentially expressed between major tissues is currently unknown. To determine relative levels of Zcchc6 and Zcchc11 mRNA in adult tissues, we assessed transcripts encoding both enzymes by qRT-PCR. As shown in Fig 1A, Zcchc6 and Zcchc11 mRNA is detectable in all tissues examined. Interestingly, when we sought to identify where Zcchc6 mRNA was significantly enriched relative to Zcchc11 expression (Zcchc6 mRNA was normalized to Zcchc11 mRNA) we found that murine lungs exhibited enhanced Zcchc6 levels thus suggesting a more prominent role for this TUT.

In an effort to determine which lung cell(s) express Zcchc6, we queried a previously published microarray dataset from our laboratory that profiled epithelial and non-epithelial cell populations isolated during pneumonia (GSE71623) [37]. Zcchc6 was 3.3 fold more abundant in non-epithelial cells than epithelial cells during pneumonia (FDR q<0.05). These data also suggested that Zcchc6 was selectively enriched over Zcchc11 in adult lungs and that non-epithelial cell sources predominated as sources of Zcchc6 expression. Hence, given previous reports that Zcchc6 and Zcchc11 have overlapping and compensatory function, we sought to determine whether Zcchc6 plays a predominant role in the myeloid compartment of the lung.

Alveolar macrophages represent a critical first line of defense against inhaled pathogens in the lungs [38–40] Given that Zcchc6 expression was enriched in non-epithelial lung populations of cells, we examined Zcchc6 protein expression in alveolar macrophages. Human alveolar macrophages collected from healthy donors by BAL contain Zcchc6 protein that is readily detectable by immunoblot. We observed that Zcchc6 protein is not expressed in peripheral blood monocytes, but is induced when these cells were differentiating during cell culture adhesion, *in vitro* (Fig 1B). Although previous reports suggest that Zcchc6 may be restricted to un-differentiated cell types [24], these findings indicate the opposite expression pattern within the myeloid lineage. To further test whether Zcchc6 protein expression results from monocyte-to-macrophage differentiation, we used the human monocytic cell lines U937 and THP-1, whose maturation can be pharmacologically induced in cell cultures [41]. As shown in Fig 1C, Zcchc6 protein expression was markedly induced upon monocyte-to-macrophage differentiation in both cell lines. Similar to the human models of macrophage differentiation, we also observed enhanced Zcchc6 protein expression in mouse bone marrow-derived macrophages during differentiation *in vitro* (Fig 1D). These multiple lines of evidence consistently demonstrated the induction of the RNA uridyltransferase Zcchc6 during the progression from monocytes to macrophage-like cells.

### Zcchc6-deficient mice are viable and born in Mendelian ratios

**Fig 2:**
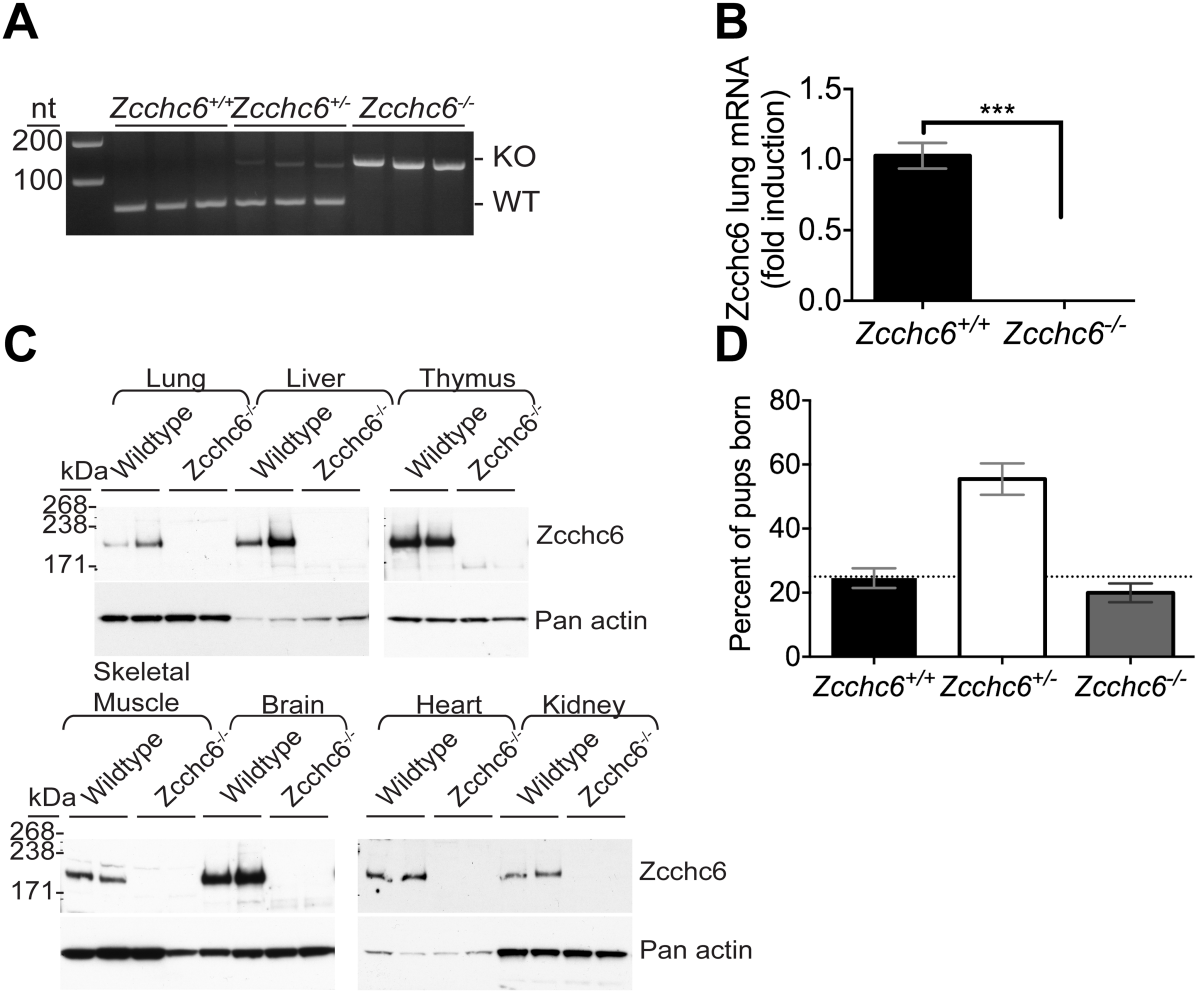
Establishment of a mouse model of Zcchc6 deficiency. (A) Gene rearrangement in Zcchc6^-/-^ mice at the DNA level was confirmed by PCR of tail genomic DNA. (B) Zcchc6 mRNA expression was measured by qRT-PCR from total left lobe lung RNA and normalized to 18S rRNA. Data expressed as mean and SEM, ***p<0.001 by Student’s t-test (C) Immunoblot analysis of Zcchc6 protein expression across multiple tissue cell lysates. (D) Genotypes of pups weaned from Zcchc6^+/-^ x Zcchc6^+/-^ crosses. Dotted line indicated expected frequency of homozygous animals.

To determine whether Zcchc6 is essential for macrophage function *in vivo*, we created Zcchc6-deficient mouse model (*Zcchc6*^*-/-*^). Genomic deletion was confirmed via PCR on genomic tail DNA samples (Fig 2A), and mRNA analysis revealed effective loss of lung expression due to mutation (Fig 2B). At the protein level, immunoblot analysis confirmed a complete loss of Zcchc6 protein expression in all tissues examined (Fig 2C). These results demonstrate that Zcchc6 protein is widely-expressed in wild type mice but eliminated in Zcchc6-targeted animals. Because of the 50% perinatal mortality observed in *Zcchc11*^*-/-*^ mice [19], pups born from breeding heterozygous *Zcchc6*^*+/-*^ × *Zcchc6*^*+/-*^ mating pairs were closely monitored. Unlike Zcchc11-deficient animals, wild type, heterozygous and homozygous *Zcchc6*^*-/-*^ mice exhibited normal litter sizes and were born in ratios consistent with Mendelian patterns of inheritance (Fig 2D). No changes in anatomy or behavior were observed as mice aged to adulthood. Taken together, our data demonstrate that genetic loss of Zcchc6 is compatible with murine life.

### Homeostatic macrophage cell number and transcriptomic profiles are not impacted by Zcchc6 deficiency

**Fig 3:**
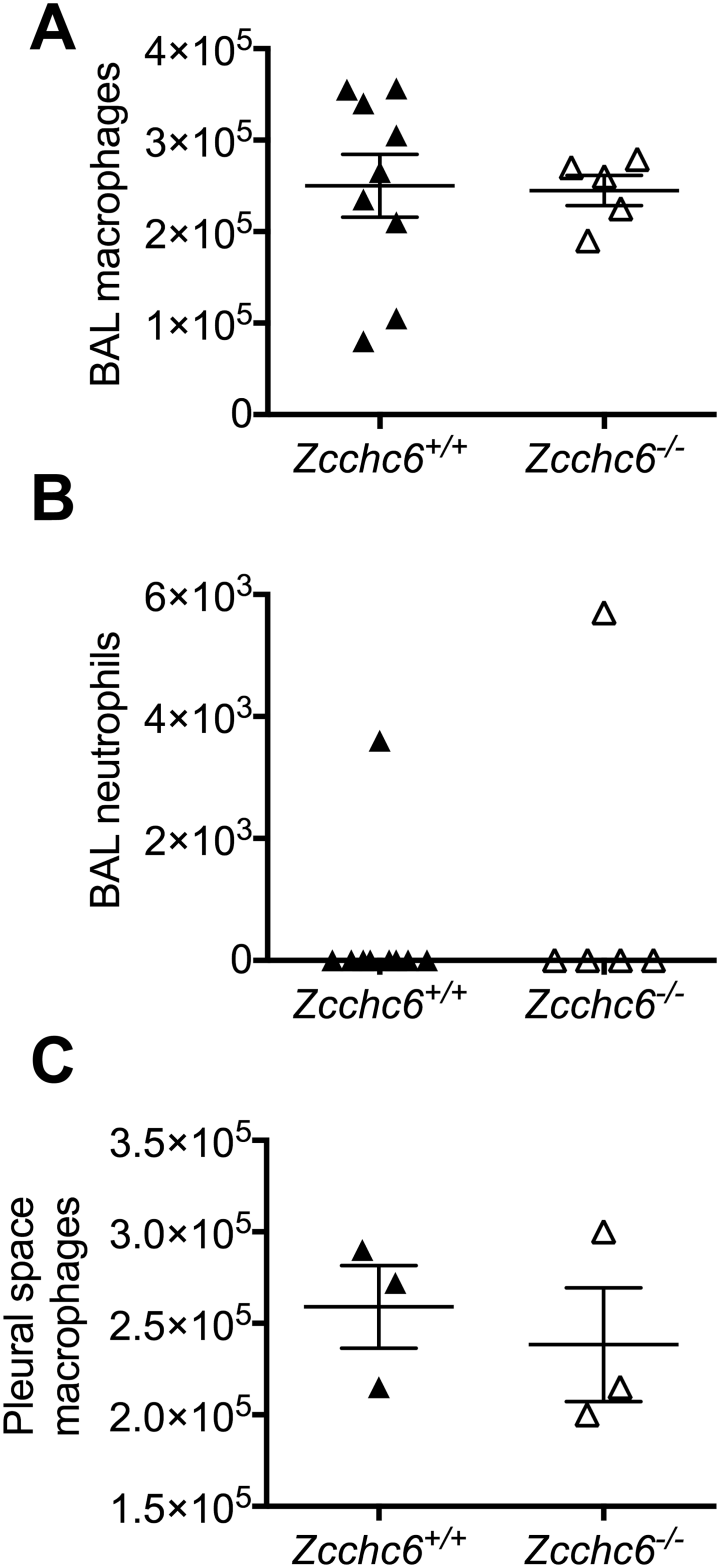
Homeostatic macrophage cell numbers are unchanged in Zcchc6-deficient mice under basal conditions. (A) Total alveolar macrophages and (B) airspace neutrophils were isolated by bronchoalveolar lavage and quantified by hemocytometer and cellular differential analysis by Diffquick staining. (C) Pleural macrophages were collected by lavage and quantified by hemocytometer. Data were determined to be statistically non-significant by Student’s t-test.

Our data demonstrate that Zcchc6 is expressed in macrophages and induced during the transition from monocytes to macrophage-like cells. Given these observations and that the Zcchc6 homologue Zcchc11 mediates cell proliferation [42], we sought to determine whether baseline homeostatic macrophage numbers were altered in the Zcchc6-deficient animals. We observed no significant differences in the number of alveolar macrophages recovered by bronchoalveolar lavage (Fig 3A), and as expected, neutrophils were rarely recoverable from uninfected mice in the presence or absence of Zcchc6 (Fig 3B). Since macrophages also reside in the pleural space, we lavaged the pleural cavity in which we found no differences in macrophage numbers between genotypes (Fig 3C).

Without evidence of a biological phenotype under basal conditions, we sought to determine whether loss of Zcchc6 would yield a molecular phenotype. To assess the role of Zcchc6 in the regulation of gene expression, we profiled BAL accessible macrophages by microarray. Total RNA was prepared from alveolar macrophages isolated from *Zcchc6*^*+/+*^ and *Zcchc6*^*-/-*^ mice. Global mRNA expression was determined using Affymetrix Mouse Gene 2.0 ST microarrays. To our surprise, we discovered that not a single mRNA was differentially expressed between genotypes to a statistically significant degree (FDR q < 0.05, GSE98222). Gene set enrichment analysis demonstrated an increase in GO Biological processes primarily associated with cell cycle regulation and DNA replication (Table 1) Collectively, these data suggest that that Zcchc6 is dispensable for the development and maintenance of the lung macrophage population, as well as basal transcriptional state.

**Table 1:** Top 20 GO Biological process terms.

Gene Set Name	Gene Set Size	Normalized Enrichment Score (NES)	FDR q value
CELL_CYCLE_PROCESS	185	2.96	***0*.*00***
M_PHASE	109	2.85	***0*.*00***
MITOTIC_CELL_CYCLE	145	2.85	***0*.*00***
CELL_CYCLE_PHASE	162	2.82	***0*.*00***
MITOSIS	79	2.78	***0*.*00***
M_PHASE_OF_MITOTIC_CELL_CYCLE	82	2.77	***0*.*00***
CELL_CYCLE_GO_0007049	299	2.61	***0*.*00***
DNA_REPAIR	120	2.52	***0*.*00***
CHROMOSOME_ORGANIZATION_AND_BIOGENESIS	116	2.48	***0*.*00***
DNA_REPLICATION	97	2.46	***0*.*00***
CHROMOSOME_SEGREGATION	30	2.45	***0*.*00***
DNA_METABOLIC_PROCESS	245	2.43	***0*.*00***
DNA_DEPENDENT_DNA_REPLICATION	53	2.41	***0*.*00***
RESPONSE_TO_DNA_DAMAGE_STIMULUS	154	2.41	***0*.*00***
SISTER_CHROMATID_SEGREGATION	16	2.35	***0*.*00***
MICROTUBULE_CYTOSKELETON_ORGANIZATION_AND_BIOGENESIS	35	2.34	***0*.*00***
CELL_CYCLE_CHECKPOINT_GO_0000075	46	2.29	***1*.*29E-05***
MITOTIC_SISTER_CHROMATID_SEGREGATION	15	2.29	***1*.*27E-05***
RESPONSE_TO_ENDOGENOUS_STIMULUS	189	2.27	***1*.*24E-05***
INTERPHASE_OF_MITOTIC_CELL_CYCLE	58	2.26	***1*.*17E-05***

### Maximal innate immune responses in macrophages require Zcchc6 during pneumonia

**Fig 4:**
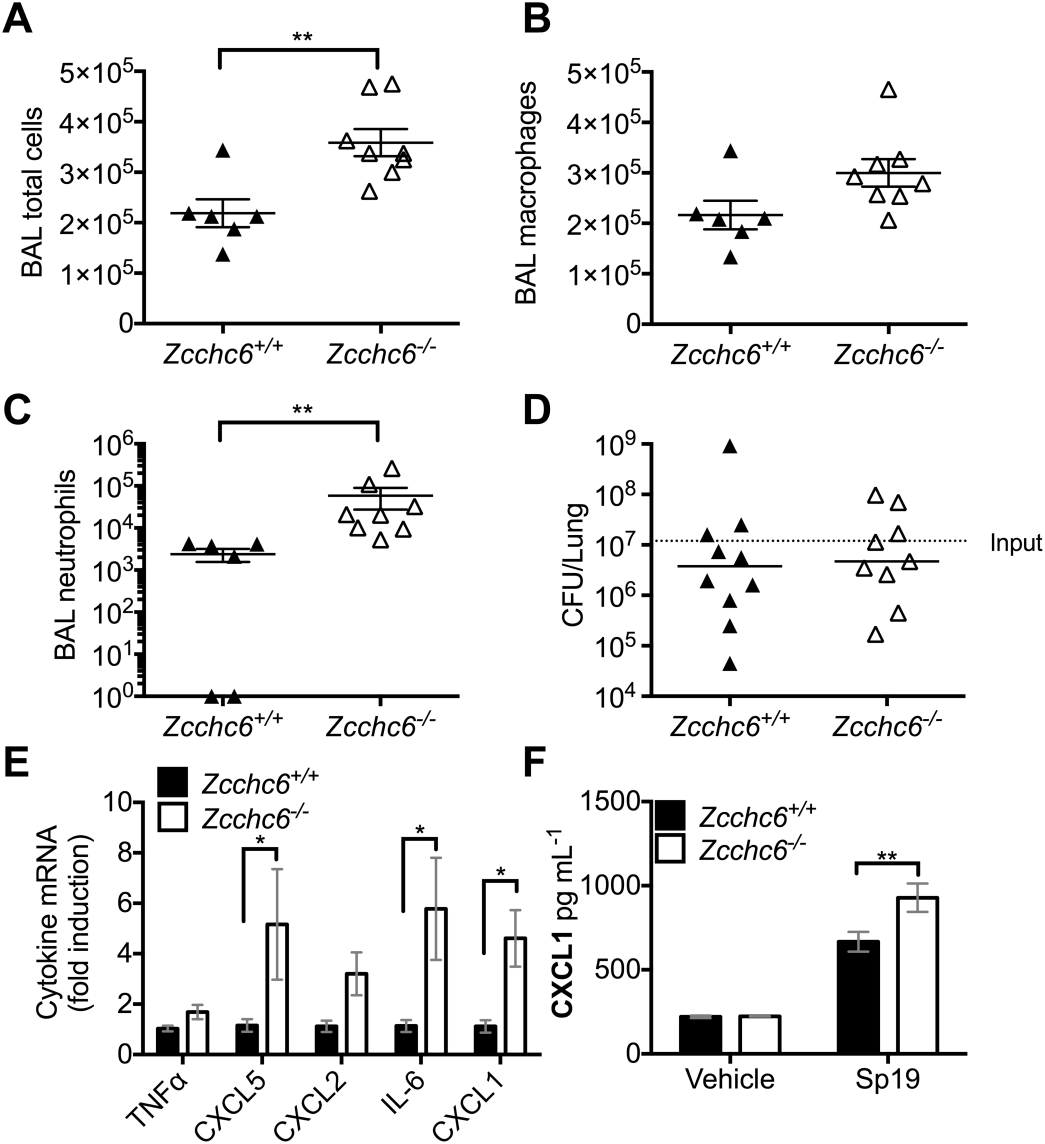
Zcchc6 minimally directs neutrophil emigration during pneumococcal pneumonia. (A) Total BAL cell counts (B) alveolar macrophage numbers and (C) neutrophil emigration were assessed by bronchoalveolar lavage from *Zcchc6*^*+/+*^ or *Zcchc6*^*-/-*^ mice infected with of Sp19 i.t. for 4 hours. **p<0.01 by Student’s t-test. (D) Bacterial lung burdens were determined from *Zcchc6*^*+/+*^ or *Zcchc6*^*-/-*^ mice 30 hours post intratracheal instillation of Sp19. (E) Cytokine mRNA expression was measured by qRT-PCR on total lung left lobe RNA isolated from Sp19-infected *Zcchc6*^*+/+*^ and *Zcchc6*^*-/-*^ mice at 4 hours post infection. Data are normalized to 18S rRNA levels and expressed as fold induction over *Zcchc6*^*+/+*^. n = 6,8; *p<0.05 by Student’s t-test. (F) CXCL1 protein levels were measured by ELISA in supernatants collected from cultured BMDMs (n = 4,3) stimulated with 1 x 10^6^ CFU/mL Sp19.

Given no observable biological or molecular phenotype of Zcchc6-deficient mice under basal conditions, we next sought to determine the influence of Zcchc6 in a setting of physiological duress, bacterial pneumonia. Host defense against bacterial lung infections is coordinated amongst macrophages and multiple other cell types [43, 44]. We intratracheally infected littermate *Zcchc6*^*+/+*^ and *Zcchc6*^*-/-*^ mice with *S*. *pneumoniae* serotype 19 (Sp19). Early host immune cell recruitment was assessed after 4 hours of infection due to the potential impact on bacterial growth over 30 hours of infection. We observed that mice deficient in Zcchc6 exhibited increased total cells recovered from the airspaces by lavage early during infection (Fig 4A). Macrophage numbers were not significantly altered (Fig 4B), however there was a significant increase in emigrated neutrophils (Fig 4C) at 4 hours after infection. Despite this difference in early neutrophil recruitment, bacterial burdens were equivalent in *Zcchc6*^*+/+*^ and *Zcchc6*^*-/-*^ mice (Fig 4D) at 30 hours after infection. To assess the potential impact of Zcchc6-deficiency on host defense, we investigated a second model of acute bacterial pneumonia. We intratracheally infected littermate *Zcchc6*^*+/+*^ and *Zcchc6*^*-/-*^ mice with the Gram-negative pathogen *E*. *coli*. After 24 hours of infection, total cell counts and BAL differentials were unchanged (S1 Fig). In addition, bacterial burdens in both the lung and circulation were equivalent in *Zcchc6*^*+/+*^ and *Zcchc6*^*-/-*^ mice (S1 Fig). Taken together with the Sp19 results, these data provide additional evidence that Zcchc6-deficiency is not sufficient to alter host defense during acute bacterial pneumonia.

To investigate potential signals upstream of enhanced neutrophil recruitment we analyzed three major murine neutrophil chemokines (CXCL1, CXCL2, and CXCL5), as well as TNF and IL-6 by qRT-PCR. Significantly enhanced induction of CXCL5, IL-6 and CXCL1 was observed in *Zcchc6*^*-/-*^ mice with similar trends in the other targets analyzed (Fig 4E). To further test whether Zcchc6 expression regulates macrophage-specific gene expression during bacterial stimulation we isolated BMDMs from *Zcchc6*^*+/+*^ or *Zcchc6*^*-/-*^ mice and exposed them directly to Sp19. Consistent with our *in vivo* findings, *S*. *pneumoniae*-induced CXCL1 protein expression was increased in the absence of Zcchc6 (Fig 4F). Collectively, these results indicate that Zcchc6 regulates macrophage innate immune responses; although, its impact on macrophage-dependent immunity was insufficient to yield differences in antibacterial defense within the current experimental circumstances.

## Discussion

Zcchc6 and Zcchc11 TUTs have been reported to regulate miRNA maturation in embryonic stem cells and cancer cell lines by oligo-uridylating let-7 family member precursor miRNAs in conjunction with Lin28a [14, 15, 21, 45, 46]. In the absence of Lin28a, both TUTs were shown to mono-uridylate group II miRNAs resulting in enhanced Dicer processing and increased mature levels of the cognate miRNA [22]. Regardless of whether Lin28 is present or absent, Zcchc6 and Zcchc11 were shown to play a key role in the post-transcriptional maturation of mature miRNA [47]. All of these results, however, were obtained from cell culture systems, and our results from mice deficient in Zcchc6 suggest that any contributions of Zcchc6 alone to embryonic stem cell biology are not essential for development. As suggested previously [48], modest or absent phenotypes due to loss of a single TUT, Zcchc6, may be due in part to overlapping and functionally compensatory roles of these TUTs *in vivo*. Future studies will be required to investigate whether double knockout models of Zcchc6 and Zcchc11 exhibit more pronounced phenotypes *in vivo*.

While previous studies indicate a role for Zcchc6, and other TUTs, in undifferentiated cells and tissues, we observed a widespread expression of Zcchc6 protein in all adult somatic tissues examined. Given potential compensatory regulation between TUTs [24, 49], we compared Zcchc6 to Zcchc11 and asked whether there were any tissues where Zcchc6 was selectively enriched over Zcchc11. In the liver and in the lungs, Zcchc6 mRNA was significantly elevated over Zcchc11 mRNA. Zcchc6 was expressed in myeloid cells in humans and mice and was induced upon the transition from monocyte to macrophage-like cells. The spatially and temporally distinct expression patterns of Zcchc6 and Zcchc11 identify select instances where overlapping roles are unlikely. The observation that Zcchc6 was increased with macrophage differentiation was unanticipated, since both Zcchc6 and Zcchc11 often mark less differentiated cells [24] and since Zcchc11 is not observed in any myeloid cells ([19] as well as data not shown). Despite its prominent expression, and the enrichment of proliferative genes, Zcchc6 deficiency did not affect alveolar macrophage numbers or transcriptional profiles under basal conditions, demonstrating this enzyme to be dispensable for macrophage differentiation.

Recent studies have shown that during stress responses there are dramatic changes in gene expression that occur independently of transcriptional control mechanisms [50, 51]. Given previous reports demonstrating TUT-dependent roles in post-transcriptional gene regulation and that Zcchc6 does not impact transcriptomic changes under basal conditions, we sought to determine whether Zcchc6-deficiency exerts any influence on gene expression under infectious stress. In contrast to homeostatic conditions, we report an increase in cytokine expression during pneumonia in the Zcchc6-deficient mice compared to wildtype controls, particularly IL-6 and CXCL1. An apparent reduction of IL-6 by Zcchc6 was especially surprising given that Zcchc11 is essential to maximal induction of this cytokine [18]. We did not observe increased expression of Zcchc11 due to Zcchc6 deficiency, so a compensatory increase in Zcchc11-mediated IL-6 expression is unlikely to explain these results. Instead, these two TUTs may exert opposing roles in the modulation of IL-6 expression. The miRNAs that target cytokine transcripts can silence expression, which applies to the Zcchc11 target miR-26 [18]. Conversely, miRNAs have been reported to enhance cytokine expression [52, 53]. The mechanism by which Zcchc6 regulates IL-6 expression remains to be determined, but could involve miRNA uridylation as has been observed for Zcchc11 [18]. Elevated macrophage cytokine expression due to Zcchc6 deficiency was associated with accelerated neutrophil recruitment during pneumonia, but this did not impact bacterial burdens in the experimental conditions tested. Regardless, Zcchc6-dependent cytokine regulation may serve as an important determinant of inflammation, immunopathology, and/or pathogen clearance under circumstances yet to be investigated. To our knowledge, the regulation of macrophage cytokines is the very first functional role for Zcchc6 that has emerged from an animal model of Zcchc6 deficiency.

Accumulating evidence has shown that nontemplated 3′ RNA tailing, in particular uridylation, is more prevalent within mammalian transcriptomes than previously thought. It was previously shown that miRNA-directed cleavage of mRNA products were uridylated [54]. Additionally, histone mRNAs were demonstrated to be uridylated and subsequently degraded at the end of the cell cycle S phase [55–58]. Recently, the development of a TAIL-Seq method provided a more comprehensive glimpse at the genome-wide assessment of mRNA 3′ tail uridylation in mammalian cells [25, 59]. The majority of mRNAs in NIH3T3 and HeLa cells are terminally uridylated and surprisingly, these uridylation signatures were detected predominantly on short polyadenylate tails [25, 59]. These results further supported the notion that mRNAs designated for and in the process of decay are flagged by 3′ terminal uridylation. In the context of infection and inflammation, host innate responses result in a dramatic upregulation of a diverse set of inflammatory mediator mRNAs. Most cytokine and chemokine transcripts are extremely labile and contain a multitude of AU-rich elements within their 3′ UTRs which could have a profound impact on the temporal order of inflammatory gene expression [60]. It is conceivable that Zcchc6-mediated addition of uridines could serve as another post-transcriptional regulatory step to prevent excessive inflammation. If so, variations in these pathways, such as by genetic or environmental alterations of Zcchc6 expression or activity, could influence susceptibility to diverse inflammatory disorders such as colitis, arthritis, and more. Future studies are required to delineate how Zcchc6 may regulate cytokine expression by macrophages in diverse inflammatory settings.

We are just beginning to understand the complex role(s) of TUT family members and their function in gene regulation and physiology in integrated animal systems. In this study we present the first mouse model of Zcchc6 deficiency. Zcchc6 does not impact development or survival through young adulthood. Notably, Zcchc6 expression is increased during the transition from monocytes to macrophage-like cells, where it contributes to cytokine regulation in response to acute stimulation. These results were observed during a model of bacterial pneumonia, and very well may be relevant to other infectious or inflammation-based disorders as well. Future studies are needed to determine if other immune-related phenotypes result from loss of Zcchc6-dependent activities. In addition, future studies based on dual deficiency of both Zcchc6 and Zcchc11 are critical to determine whether embryonic development or other integrated biological systems in living animals specifically requires TUT-dependent biology.

## Supporting information

S1 Fig Zcchc6 deficiency does not impact the host response to Gram-negative pneumonia. (A) Total BAL cell counts (B) airspace cell differentials (C) blood CFU and (D) lung CFU were assessed in *Zcchc6*^*+/+*^ or *Zcchc6*^*-/-*^ mice infected with of 2 x 10^6^ CFU *E*. *coli* i.t. for 24 hours. Data were determined to be statistically- non significant by Student’s t-test (A and B) or Mann Whitney test (C and D).
(TIF)
