# Supplementary figures and images for: The RNA uridyltransferase Zcchc6 is expressed in macrophages and impacts innate immune responses
Source: PLoS One. 2017 Jun 30;12(6):e0179797. doi: 10.1371/journal.pone.0179797 (PMC5493306; doi:10.1371/journal.pone.0179797)

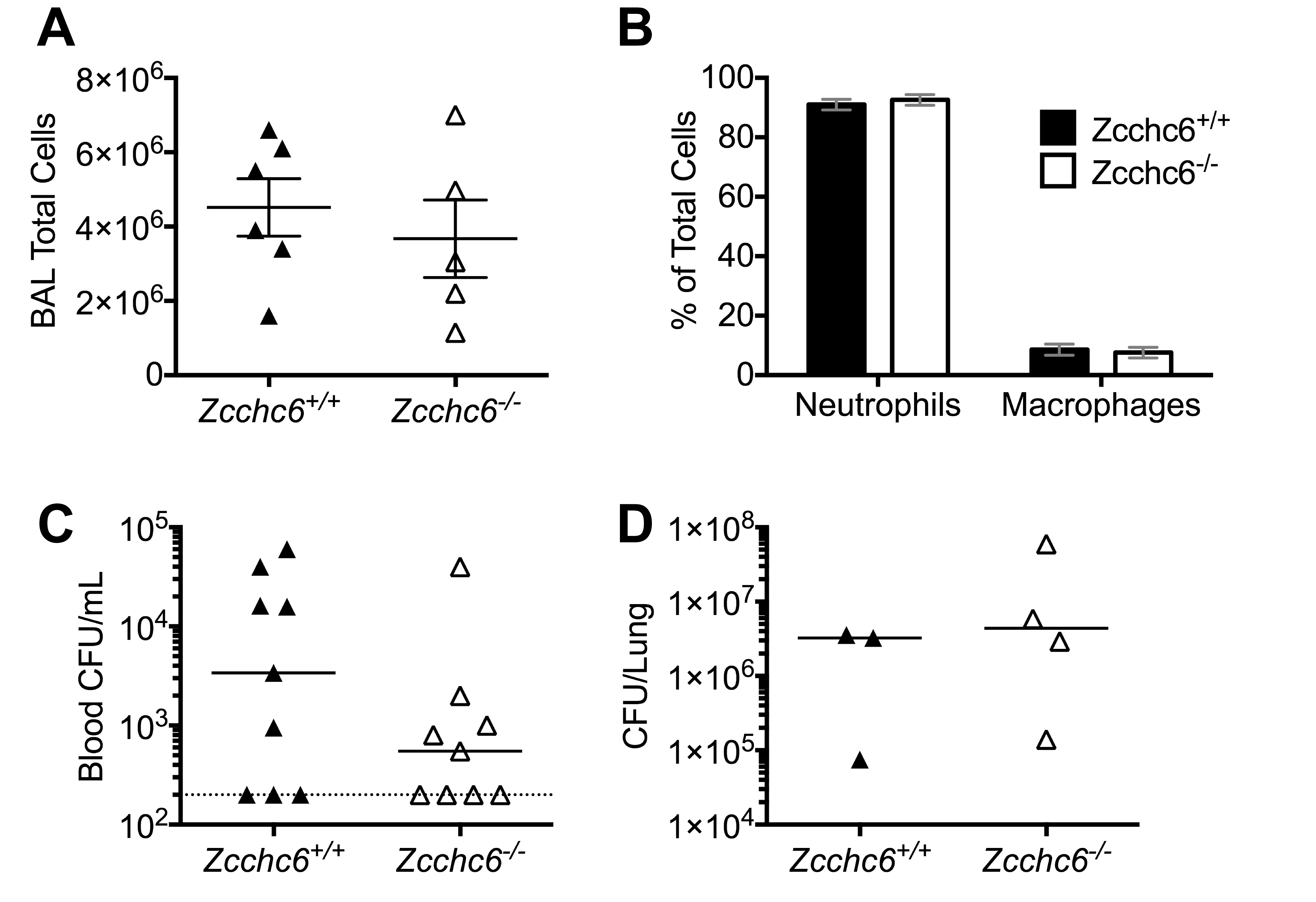

Supplement: S1 Fig — (A) Total BAL cell counts (B) airspace cell differentials (C) blood CFU and (D) lung CFU were assessed in Zcchc6+/+ or Zcchc6-/- mice infected with of 2 x 10^6^ CFU E. coli i.t. for 24 hours. Data were determined to be statistically- non significant by Student’s t-test (A and B) or Mann Whitney test (C and D). (TIF) [file pone.0179797.s001.tif]
